# Implementation of a patient participation strategy in a randomized controlled hand hygiene promotion study – a mixed-method qualitative and quantitative evaluation

**DOI:** 10.1186/1753-6561-5-S6-P266

**Published:** 2011-06-29

**Authors:** S Touveneau, A Stewardson, M Schindler, W Zingg, M Bourrier, D Pittet, H Sax

**Affiliations:** 1 Infection control program, University of Geneva Hospitals, Geneva, Switzerland; 2 Departement of Sociology, University of Geneva, Geneva, Switzerland

## Introduction / objectives

An ongoing, ward-level cluster-randomized, controlled, multimodal hand hygiene (HH) promotion study includes a patient participation component (PP): patients and healthcare workers (HCW) remind each other of HH after personal invitation by HCW using an information pack (IP).

## Methods

During implementation we interviewed the head nurses of the 19 PP wards at months 3 and 6. Their beliefs about PP, perception of the ward’s uptake, promotional engagement, perception of facilitators and barriers, and corrective actions were discussed. Interview notes were analyzed inductively and independently by 2 investigators. Beliefs, uptake perception, and engagement were quantified on a 5-point scale. The proportion of patients receiving IP was used as a measure of ward engagement.

## Results

Mean IP distribution incidence was 36% (SD, ±30). Mean scores for beliefs, uptake perception, and engagement were 3.58 (±1.0), 3.9 (±0.6), and 3.55 (±1.0), respectively, with a negative trend. Qualitative analysis identified the following barriers: HCW perception of unsuitable patient profiles, HCW fear of patient reactions, competing projects, high workload, HCW and patient turnover, and general work climate issues. Positive deviant conditions were: assistant nurse involvement, identifying suitable patients during handovers, and communication training of HCW with patients. We identified 2 outlier champion wards.

## Conclusion

Implementation is slow in this challenging social behavior project. The mixed-method approach allowed efficient identification of the main issues and amendment opportunities.

## Disclosure of interest

None declared.

